# Complete Genome Sequence of *Acidianus* sp. Strain HS-5, Isolated from the Unzen Hot Spring in Japan

**DOI:** 10.1128/mra.01159-21

**Published:** 2022-02-17

**Authors:** Hiromi Omokawa, Norio Kurosawa, Hiroyuki D. Sakai

**Affiliations:** a Department of Environmental Engineering for Symbiosis, Graduate School of Science and Engineering, Soka University, Hachioji, Tokyo, Japan; b Department of Science and Engineering for Sustainable Innovation, Faculty of Science and Engineering, Soka University, Hachioji, Tokyo, Japan; Indiana University, Bloomington

## Abstract

The genus *Acidianus* is composed of facultatively aerobic archaea growing on elemental sulfur as an energy source. Here, we report the 2.58-Mb complete genome sequence of *Acidianus* sp. strain HS-5, which was isolated from a sulfur hot spring located in Unzen, Japan.

## ANNOUNCEMENT

The genus *Acidianus*, belonging to the order *Sulfolobales*, is one of the major taxa often dominating in acidic geothermal environments (1). Currently, four species have been validly described in this genus, namely, Acidianus brierleyi, Acidianus infernus, Acidianus ambivalens, and Acidianus sulfidivorans (2–4). The species are typically known as chemolithotrophs, utilizing elemental sulfur as an electron donor or acceptor under aerobic or anaerobic conditions, respectively. We isolated another novel strain, HS-5, which should belong to the genus *Acidianus*. Here, we report the complete genome sequence of strain HS-5.

Muddy water was collected at a sulfur hot spring located in Unzen, Japan, as described previously (5). An aliquot of the sample was inoculated into modified Brock’s basal salt medium (6) supplemented with 0.5 g/L peptone, and then enrichment culture was conducted at 65°C. The pH of the medium was adjusted to 1.5 by adding 50% H_2_SO_4_. Strain HS-5 was isolated from this enrichment culture by the dilution-to-extinction method. Genomic DNA was extracted from 1 L of the culture using Genomic-tips 100/G (Qiagen). For short-read sequencing, a DNA library was constructed using the NEBNext Ultra II FS DNA library preparation kit for Illumina (New England Biolabs). Short-read sequencing was conducted using the Illumina NovaSeq 6000 platform (2 × 150 bp). Long-read sequencing was carried out on a MinION sequencer with an R9 flow cell, SQK-LSK109, and EXP-NBD104 (Oxford Nanopore Technologies), following the protocol described by Oxford Nanopore Technologies (NBE_9065_v109_revZ_14Aug2019). DNA fragments of 3 kb or longer were enriched by the protocol. Base calling was performed by MinKNOW v.4.2.8 software. A total of 11,689,538 short reads (1,753,430,700 bp) and 74,540 long reads (560,714,431 bp [*N*_50_, 13,421 bp]) were obtained from Illumina NovaSeq 6000 and MinION sequencers, respectively. The short reads were quality filtered using Fastp v.0.20.1 (7), resulting in a total of 11,651,978 quality-filtered reads (1,694,561,488 bp). The long reads were quality filtered using Filtlong v.0.2.0 (https://github.com/rrwick/Filtlong), resulting in a total of 96,304 quality-filtered long reads (509,939,913 bp [*N*_50_, 8,059 bp]). The quality-filtered reads were used for genome assembly by Unicycler v.0.4.8 (8), followed by annotation with DFAST v.1.4.0 (9). Default parameters were used for all software.

**FIG 1:**
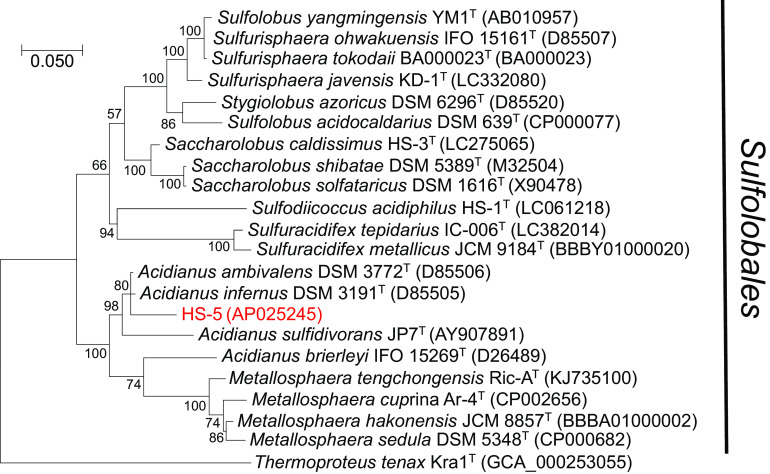
Maximum likelihood phylogenetic tree of 16S rRNA gene sequences. The sequence alignment was performed by the MUSCLE program implemented in MEGA X (10), with default parameters. Bootstrap values derived from 1,000 samplings are indicated at the branch nodes. GenBank accession numbers are indicated in parentheses.

A circular contig of 2,584,028 bp, with a GC content of 35.1%, was obtained. The genome contained 2,853 coding sequences (CDSs), a single copy of the rRNA operon, and 46 tRNAs. The maximum likelihood tree of the 16S rRNA gene constructed by MEGA X (10) showed that strain HS-5 clustered with members of the genus *Acidianus* except for A. brierleyi (Fig. 1). The BLASTN search (11) of the 16S rRNA gene sequence against the GenBank nonredundant database showed that the closest species to strain HS-5 was A. ambivalens, with 96.9% 16S rRNA sequence similarity. This value was significantly lower than the species threshold value of 98.65% (12), suggesting that HS-5 represents a novel species of the genus *Acidianus*. After the Kyoto Encyclopedia of Genes and Genomes (KEGG) ortholog numbers for all CDSs were retrieved using BlastKOALA (13) and KofamKOALA (14), the metabolic pathways of strain HS-5 were predicted using the KEGG Mapper (15). Strain HS-5 has a complete set of genes encoding the tricarboxylic acid cycle, the archaeal pentose phosphate pathway, and the 3-hydroxypropionate/4-hydroxybutyrate cycle. In addition, genes associated with sulfur metabolism, such as *phsA*, *doxAD*, *sor*, *sqr*, *sreABCDE*, and *soeB*, were found in the genome, indicating that strain HS-5 may be involved in the sulfur cycle in its habitat.

### Data availability.

The genome sequence of strain HS-5 and the raw reads have been deposited in DDBJ/ENA/GenBank under the accession numbers AP025245, DRR325693, and DRR325694.
